# Characterizing the structural properties and porosity of mid-urethral slings with varied manufacturing techniques

**DOI:** 10.3389/fbioe.2025.1543808

**Published:** 2025-08-07

**Authors:** Katrina Knight, Leslie Meyn, Pamela Moalli

**Affiliations:** ^1^ Department of Bioengineering, University of Pittsburgh, Pittsburgh, PA, United States; ^2^ Magee-Womens Research Institute, Pittsburgh, PA, United States; ^3^ Department of Obstetrics, Gynecology, and Reproductive Sciences, University of Pittsburgh, Pittsburgh, PA, United States

**Keywords:** mid-urethral sling, incontinence, porosity, permanent elongation, polypropylene mesh, cyclical loading

## Abstract

**Introduction:**

Implantation of mid-urethral slings (MUSs) is a safe and effective approach for the surgical repair of stress urinary incontinence. However, concerns regarding the deformability of the prototype MUS, mechanical cut Gynecare TVT prompted manufacturers to use techniques like laser cutting, heat-sealing, and the inclusion of an interwoven stabilizing suture to decrease deformation with loading. We hypothesized that a laser cut or heat-sealed MUS would be stiffer but deform less, and experience less permanent elongation as compared to a mechanical cut MUS. Additionally, the inclusion of a stabilizing suture would minimize the loss of porosity.

**Methods:**

Uniaxial tensile testing to failure and cyclic loading was performed to analyze the structural properties and permanent elongation, respectively, of commercially available MUSs Gynecare TVT (mechanical cut), Gynecare TVT Exact (laser cut), ArcTV (laser cut, with and without the stabilizing suture), and Desara Blue (heat-sealed). A custom Mathematica code was used to quantify the porosity of the MUSs following sequential uniaxial loading from 0 N to 10 N.

**Results:**

Desara Blue was significantly stiffer (p-values < 0.05), elongated less at failure (p = 0.002), and experienced less permanent elongation in response to cyclic loading (p-values = 0.001) relative to Gynecare TVT. Similarly, permanent elongation was significantly less (p-values = 0.004) and the stiffness was higher (p = 0.004) for Gynecare TVT Exact as compared to Gynecare TVT. Very little differences in stiffness and no differences in relative elongation at failure nor permanent elongation were observed between ArcTV (without the interwoven suture) and Gynecare TVT (p-values > 0.05). The porosity of all MUSs significantly decreased with loading (p-values < 0.001); except for ArcTV with the stabilizing suture which showed the least amount of deformation (i.e., percent change in porosity decreasing by only 14%, p < 0.001, at 10 N).

**Discussion:**

Overall, heat-sealing decreased deformability at the cost of markedly increasing device stiffness to a point which likely outweighs benefits, and risks increased complications. Laser cutting had different effects on the behavior of TVT Exact and ArcTV suggesting manufacturer technical differences, but overall reduced deformation without a substantial impact on stiffness. An interwoven stabilizing suture minimized the loss of porosity which translates clinically to less deformation and mechanistically to reduced mesh complications.

## 1 Introduction

Stress urinary incontinence (SUI) is defined as involuntary leakage of urine with coughing, sneezing, or physical exertion when intraabdominal pressure exceeds urethral pressure (Luber, 2004). The pathophysiology of SUI is attributed to urethral hypermobility due to a loss of pelvic floor musculature or vaginal connective tissue support which prevents the urethra and bladder neck from sufficiently closing in response to increases in intraabdominal pressure (Wu, 2021). Similarly, a loss of urethral smooth muscle in the case of intrinsic urethral sphincter deficiency can also lead to insufficient urethral closure and SUI. To overcome the loss of support and hence restore urethral closure, non-surgical (e.g., weight loss, pelvic floor muscle training, vaginal devices, medications) and surgical interventions (e.g., Burch Colposuspension, urethral bulking agent, pubovaginal sling, mid-urethral sling) are performed (Wu, 2021). Overall, SUI is a prevalent condition among women. In the United States, the prevalence of stress urinary incontinence, when defined as any symptoms in the previous year among adult women, is approximately 46% (Wu, 2021; Abufaraj et al., 2021). Among women living in mainland China, roughly 24.5% of them have SUI while 33% and 35% of women living in Saudi Arabia and Africa, respectively, have SUI (Li et al., 2024; Alhamoud et al., 2024; Bertui et al., 2025). Similar rates of SUI have been reported for women living in France (31%), Germany (40%), Spain (39%), and the United Kingdom (41%) (Hunskaar et al., 2004). Increasing age is a predominant risk factor as well as race/ethnicity with White women having higher prevalence than Black or Hispanic women (Townsend et al., 2010). Additional risk factors include but are not limited to pregnancy, vaginal delivery, and BMI (Wu, 2021; Abufaraj et al., 2021; Tähtinen et al., 2016; Sawaqed et al., 2020).

The most commonly performed procedure for SUI treatment is the surgical implantation of a mid-urethral sling, MUS (Jonsson et al., 2012) - a minimally invasive procedure performed under intravenous sedation in which a polypropylene mesh is placed under the urethra at its mid-portion. MUSs provide a long-term solution for SUI by restoring the normal urethral support and urethral closure pressure to maintain continence during increases in intra-abdominal pressure. MUSs have become popular due to low complication rates and high cure rates. These devices can be placed via three routes - retropubic, transobturator, or single incision; however, the retropubic approach has been shown to be advantageous with a lower rate of re-operation at >5 years as compared to the transobturator route despite overall similar subjective cure rates (Ford et al., 2017; Tulokas et al., 2020; Gurol-Urganci et al., 2018). In the short-term (at 12 months), single-incision slings have been shown to be as effective as transobturator slings and may be as effective as retropubic slings for subjective cure or improvement of SUI (Carter et al., 2023). The use of MUSs has been questioned by some regulatory bodies because of complications–mainly mesh exposure through the vaginal epithelium and long-term pain. Rates of sling exposures are low, estimated at 2%–4% (Ford et al., 2017; Gurol-Urganci et al., 2018; Dejene et al., 2022; Abouassaly et al., 2004), but because symptoms tend to persist even after the sling has been removed (Pace et al., 2021), they remain problematic.

The original retropubic MUS is the Gynecare TVT (tension-free vaginal tape procedure, Ethicon–Johnson & Johnson) introduced by Ulmsten et al., in 1995 (Ulmsten et al., 1996). In this procedure, the sling, a heavy weight polypropylene mesh (80 g/m^2^), is placed under the urethra and passed through the retropubic space exiting through two incisions roughly 2 cm from mid-line immediately above the pubic rami. Based on the rapid adoption by surgeons and high success rates of the TVT, other manufacturers began creating their own variations of the retropubic sling system. Ultimately this resulted in numerous MUS systems on the market containing varied meshes; thus, making it difficult for surgeons to choose a product. In our previous studies, we suggested that the type of mesh used for MUSs may impact treatment outcomes and complications (Moalli et al., 2008). Based on synthetic biomaterials used in other soft tissue applications, we hypothesized that materials with properties (particularly stiffness) better matched to those of the tissue in which they are implanted will have fewer complications. However, at the same time, we have mechanistically demonstrated that deformations after loading in prolapse repair mesh, specifically pore collapse and wrinkling, are one of the primary drivers of prolapse mesh complications and greatly impacts the host response and downstream outcomes (Knight et al., 2022). Thus, while it is favorable to have a low weight, high porosity, low stiffness device, it may be more beneficial if the device has a stable pore geometry in response to the conditions in which it is being loaded. Generally, devices that are heavier and stiffer have more stable geometries, but this may be at the expense of increased complications.

One of the difficulties in using the Gynecare TVT is that it easily deforms with minimal manipulation. The edges of the device are also mechanically cut. Thus, when over tensioned, the free fibers generated from cutting across a knotted region, can be released into the adjacent tissue eliciting a separate foreign body response. In addition, slight over tensioning leads to permanent deformation or contraction of the mesh, associated with collapsed pores and wrinkling. To address these shortcomings, several manufacturers began sealing the edges of the mesh either by the application of heat (Advantage fit by Boston Scientific, Desara by Caldera) or individually laser sealing each disrupted knot (Gynecare TVT Exact originally by Ethicon, ArcTV by UroCure). Still others have woven a suture into the longitudinal axis to further counteract deformation without significantly altering structural properties (ArcTV). In the current study, we aimed to define the impact of manufacturing techniques on the deformability of MUSs by assessing mechanics and porosity. To do this, we performed uniaxial tensile testing to failure to obtain the structural properties of the MUSs with a particular interest in stiffness–a measure of the resistance to deformation–and the elongation at failure. Secondly, to assess how much the MUS would permanently elongate in response to repetitive cycles of loading and unloading, a cyclic loading protocol was utilized. Lastly, sequential uniaxial loading from 0 N to 10 N was done to assess how the porosity of the MUSs changes with loading. We compared Gynecare TVT Exact (laser cut edges), ArcTV (laser cut edges and a stabilizing suture), and Desara Blue (heat sealed edges) to the original mechanical cut TVT. To isolate the impact of heat sealing the edges, a secondary analysis was performed by comparing the structural properties, permanent elongation, and porosity of Desara Blue with the edges heat sealed (commercially available) to Desara Blue with the edges mechanically cut (not commercially available). We hypothesized that while laser cutting and heat sealing would be associated with reduced deformation (particularly reduced elongation at failure and permanent elongation) as compared to mechanical cut, it would substantially stiffen the MUS. Additionally, we proposed that the inclusion of a stabilizing suture would help to maintain the original MUS (pore) geometry as evidenced by a minimal change in porosity with uniaxial loading. We propose the ideal edge modification would stabilize the geometry (less deformation) while not substantially increasing MUS stiffness.

## 2 Materials and methods

### 2.1 Mid-urethral slings

**FIGURE 1:**
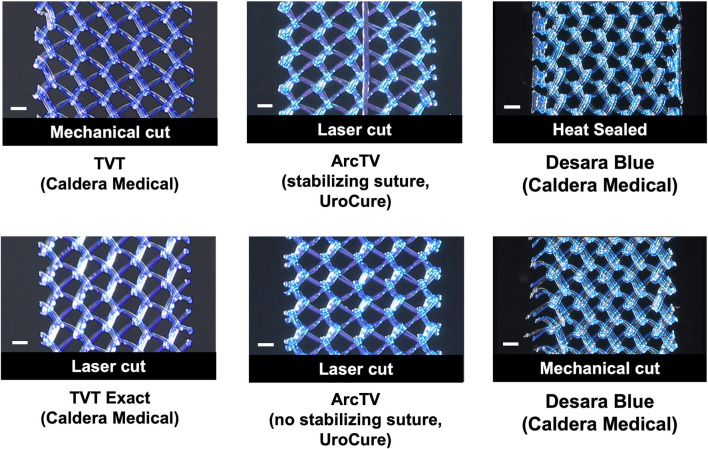
Close-up images of the mid-section of the mid-urethral slings taken with an EOS Rebel T3 camera. Scale bar represents 1 mm.

The following commercially available MUSs were analyzed in this study: Gynecare TVT (n = 6, acquired in March 2025 by Caldera Medical, Westlake Village, CA), Gynecare TVT Exact (n = 5, acquired in March 2025 by Caldera Medical, Westlake Village, CA), ArcTV (n = 14, UroCure, Minneapolis, MN), and Desara Blue (n = 14, Caldera Medical, Westlake Village, CA) (Figure 1; Table 1). They are all knitted and manufactured from polypropylene monofilament using proprietary methods. Gynecare TVT (approved via the 510(k) mechanism in 1998) is the predecessor to Gynecare TVT Exact (approved via the 510(k) mechanism in 2010). Thus, the mesh portion of Gynecare TVT Exact consists of the same knitted material and dimensions as that of Gynecare TVT. However, Gynecare TVT is mechanical cut leaving the edges free (i.e., tanged edges) versus Gynecare TVT Exact which is laser cut resulting in individually sealed edges (i.e., de-tanged edges). The ArcTV (laser cut, de-tanged edges) contains a stabilizing suture that is made of 3–0 vicryl and is secured by knots within the sling at four distinct points (center knots, two in total, placed 4.5 cm on either side of midline and one knot placed at each end of the sling). The stabilizing suture is intended to minimize the overall distortion of the sling and to prevent pore collapse during placement, tensioning, sheath removal, and during any final sling adjustments. The suture will remain implanted with the sling and will hold its strength for approximately seven to 10 days. For mechanical testing (i.e., tensile testing and the assessment of permanent elongation) the stabilizing suture was removed as we have previously shown it to have negligible contribution to the structural properties of a sling and it is designed to degrade rapidly (therefore the properties of the suture will also degrade rapidly) (Moalli et al., 2008). However, for porosity testing, ArcTV was tested with (n = 7) and without the stabilizing suture (n = 7) to assess the ability of the suture to maintain the geometry of the pores with loading. To assess the impact of applying heat to the edges of the sling, two different forms of Desara Blue mid-urethral sling were analyzed: Desara Blue heat sealed (n = 7) and Desara Blue mechanical cut (n = 7, not commercially available). It is noteworthy that heat-sealing melts the entire edge of the mesh forming thick segments along the length of the sling as opposed to laser sealing which is a more precise method of sealing the edge. Similar to Gynecare TVT, the edges of Desara Blue mechanical cut were tanged (Figure 1). Magnified images of the pores and edges of the MUSs were taken with a Jeol JSM-6390LV Scanning Electron Microscope (Jeol United States, Inc., Peabody, MA). Prior to testing, all mid-urethral slings were removed from the original packaging and protective sheath, when applicable. Overall, the manufacturing (i.e., knit pattern and pore geometry) and material were consistent along the length of all MUSs; therefore, the mid-portion of the MUS was used for tensile load-to-failure testing, while the areas proximal and distal were used for the assessment of permanent elongation and porosity.

**TABLE 1:** Company reported properties.

	Thickness (mm)	Pore size (mm)	Weight (g/m^2^)	Processing method	Mesh edges/features
TVT	0.63	1.379	100	Mechanical cut	Tanged
TVT Exact	0.63	Not reported^a^	Not reported^a^	Laser cut	De-tanged
ArcTV Sling	0.66	1.15	90	Laser cut	De-tanged
Desara Blue (heat sealed)	0.69	>1	103	Heat sealed	De-tanged
Desara Blue (mechanical cut)^b^	0.69	Not reported^a^	Not reported^a^	Mechanical cut	Tanged

^a^ It is assumed that TVT and TVT Exact and that Desara Blue heat sealed and Desara Blue mechanical cut have the same thickness, pore size, and weight.

^b^ Desara Blue (mechanical cut) is not commercially available; therefore, limited textile properties are reported by the company.

### 2.2 Tensile testing

The structural properties of the mid-urethral slings were assessed using tensile load to failure testing as previously described (Moalli et al., 2008). Briefly, samples approximately 9–10 cm long were cut from the mid-portion of each MUS and clamped on opposing ends using custom clamps. Samples were clamped such that the aspect ratio (clamp-to-clamp distance by sample width) was at least 8. Clamped samples were then submerged in a 37°C saline bath that was attached to an Instron™ 5565 testing machine such that the inferior clamp was fixed to the bottom of the saline bath (essentially at the base of the Instron™) while the superior clamp was attached to a load cell (Model 31, Honeywell, Columbus, Ohio) in series with the crosshead of the Instron™. After equilibrating in the bath for 5 min, a 0.1 N preload was applied (at 10 mm/min) and the sample was then loaded to failure at 50 mm/min. The resulting load (N) and displacement (mm) were recorded and the following structural properties were determined as previously described (Moalli et al., 2008): low stiffness (N/mm) – minimum slope over a 15% interval of relative elongation, high stiffness (N/mm) – maximum slope over a 30% interval of relative elongation, load at failure (N), relative elongation at failure (%), and relative elongation at the inflection point (%) – calculated as the intercept of two tangent lines fit to the low stiffness and high stiffness regions of the load-relative elongation curve.

### 2.3 Permanent elongation

*In vivo* MUSs will undergo repetitive cycles of loading and unloading (i.e., cyclic loading) in result of fluctuations in intra-abdominal pressure with normal physiologic functions such as sneezing, coughing, lifting, and rising from a seated position. Since MUSs are not elastic materials, they will experience unrecoverable lengthening (i.e., permanent elongation) in response to cyclic loading. To assess the amount of permanent elongation, cyclical loading was performed as described previously (Moalli et al., 2008). Briefly, the MUSs were cut to size, clamped (aspect ratio of at least 8), and submerged in a 37°C saline bath attached to the Instron as described in the tensile testing protocol Section 2.2. Samples were allowed to equilibrate for 5 min and then were preloaded to 0.1 N (at 10 mm/min). The clamp-to-clamp distance was recorded (representative of the initial length of the MUS) and the samples were subjected to the following three cyclic loading protocols: 1) Cycle 1–0.5 N–5 N for 10 cycles at 50 mm/min, 2) Cycle 2–0.5 N–15 N for 10 cycles at 50 mm/min, and 3) Cycle 3–0.5 N–5 N for 10 cycles at 50 mm/min. The forces used in this protocol are estimates of *in vivo* loading of MUSs based on the reported surface area of the urethra and urethral pressure (Diokno et al., 1991; Feng et al., 2025; Howard et al., 2000; Kapoor et al., 2012; Meng et al., 2020). Given the proximity of the urethra and vagina, the surface area of the anterior wall of the vagina and intraabdominal pressure were also used to estimate *in vivo* loading of MUSs (Howard et al., 2000; Luo et al., 2016). Thus, 5 N and 15 N were found to be within range of the forces associated with pressures measured during a range of activities (e.g., standing, coughing, valsalva), 15 N is towards the upper limit. After each Cycle, samples were preloaded to 0.1 N (at 10 mm/min) and the clamp-to-clamp distance was recorded. The amount of permanent elongation (reported as a percentage) was determined by first subtracting the initial clamp-to-clamp distance from the clamp-to-clamp distance following each cycle and then normalizing this value by the initial clamp-to-clamp distance.

### 2.4 Porosity assessment

Change in porosity with loading was assessed as previously described (Barone et al., 2016). Briefly, slings were cut, clamped (aspect ratio of at least 8), and attached to the Instron™ as described in the tensile testing protocol, Section 2.2. To prevent distortion, samples were not tested in a saline bath. Initially, slack was removed from the samples by manually lengthening the sample using the crosshead controller. Samples were then sequentially loaded to 0.1 N (rate of 10 mm/min), 5 N (rate of 50 mm/min), and 10 N (rate of 50 mm/min). The loads of 5 N and 10 N are within the physiologic range as described in Section 2.3 Permanent Elongation. After each load (including at 0 N), the mid-section of the sample was imaged using a digital SLR camera (EOS Rebel T3; Canon, Melville, NY) equipped with an EF-S 60-mm f/2.8 macro lens. All images were taken with the same lighting conditions and camera settings (high aperture, F16, and low ISO settings, ISO 100). For porosity analysis, mid-section pictures were cropped such that the only black space in the image was the space between the pores of the MUSs. The height of the cropped image was 10 mm whereas the width of the image varied depending on the width of the MUS following the application of each load. Cropped images were then imported into a custom Mathematica script and the pore size (i.e., average minimal and maximal pore diameter) and porosity (i.e., percentage of void space) were calculated for each image as well as the percent change using the porosity at 0 N as the reference. Since pore size is also an indication of void space and there is no minimal pore size established for MUSs, only the porosity data is reported in the main text of this manuscript.

### 2.5 Statistical analysis

The sample sizes chosen in this study were based on a previous study in which it was estimated that five samples per MUS were needed to detect a minimum difference of 100% in low stiffness, 15% in the relative elongation at the inflection point, and 75% difference in permanent elongation between Gynecare TVT midurethral slings and various brands with 80% power (Moalli et al., 2008). Thus, a minimal of five samples per MUS per mechanical test was analyzed. Kruskal-Wallis followed by post-hoc pairwise Mann-Whitney U tests with a Bonferroni correction (p-values<0.0167 for significance) was used to assess differences in the structural properties and permanent elongation following tensile testing and cyclic loading (respectively) between Gynecare TVT, Gynecare TVT Exact, ArcTV, and Desara Blue heat sealed. Desara Blue mechanical cut is not commercially available; therefore, Desara Blue mechanical cut was not compared to all slings but was only compared to Desara Blue heat sealed via Mann-Whitney U tests to assess the effect of heat treating the edges on the structural properties obtained following tensile testing and the porosity assessment. The overall porosity and percent change in porosity was compared using mixed effects general linear models. Pairwise comparisons were made using postestimations for the difference between coefficients estimated by the parent model, with statistical inference based on the z-statistic. Statistical analyses were performed using SPSS 28.0 (IBM, Armonk, NY) and STATA 18 (StataCorp LLC, College Station, TX). Significance was set to p < 0.05 unless otherwise stated.

## 3 Results

Since the mechanical cut Gynecare TVT (TVT) is considered the prototype mid-urethral sling, the overall pore geometry, structural properties following tensile testing, permanent elongation in response to cyclic loading, and porosity in response to tensile loading for the newer generation mid-urethral slings were compared to it. For tensile testing and permanent elongation assessment, the stabilizing suture for ArcTV was removed as it fails early and does not contribute to the structural properties.

### 3.1 Mid-urethral sling morphology

**FIGURE 2:**
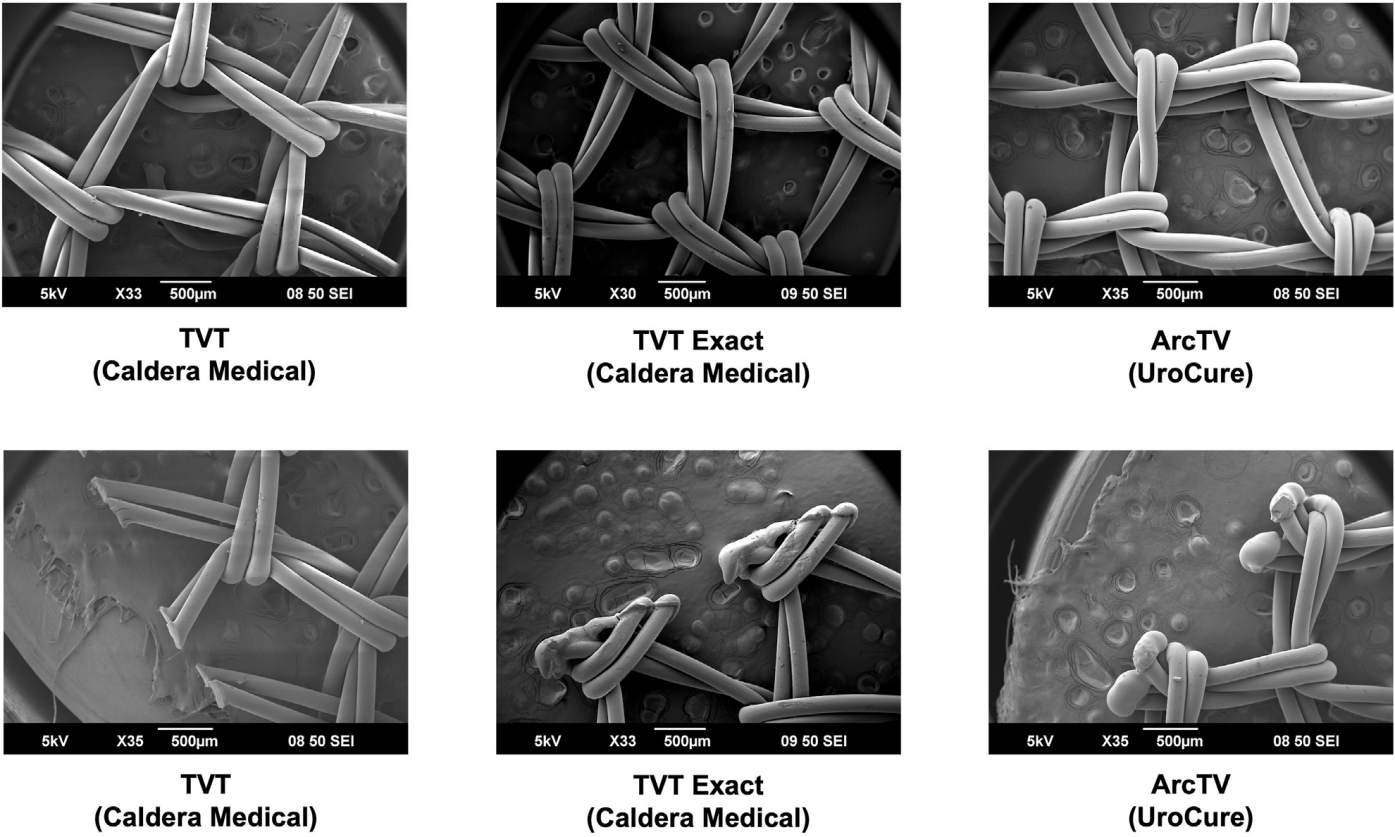
SEM images of TVT, TVT Exact, and ArcTV with complete pores shown in the top images and the edges of the respective MUSs depicted in the bottom images. Overall, the MUSs have a general pore shape that is quadrilateral. The edges of TVT (mechanical cut) are free whereas those of TVT Exact and ArcTV (both laser cut) are sealed.

**FIGURE 3 F3:**
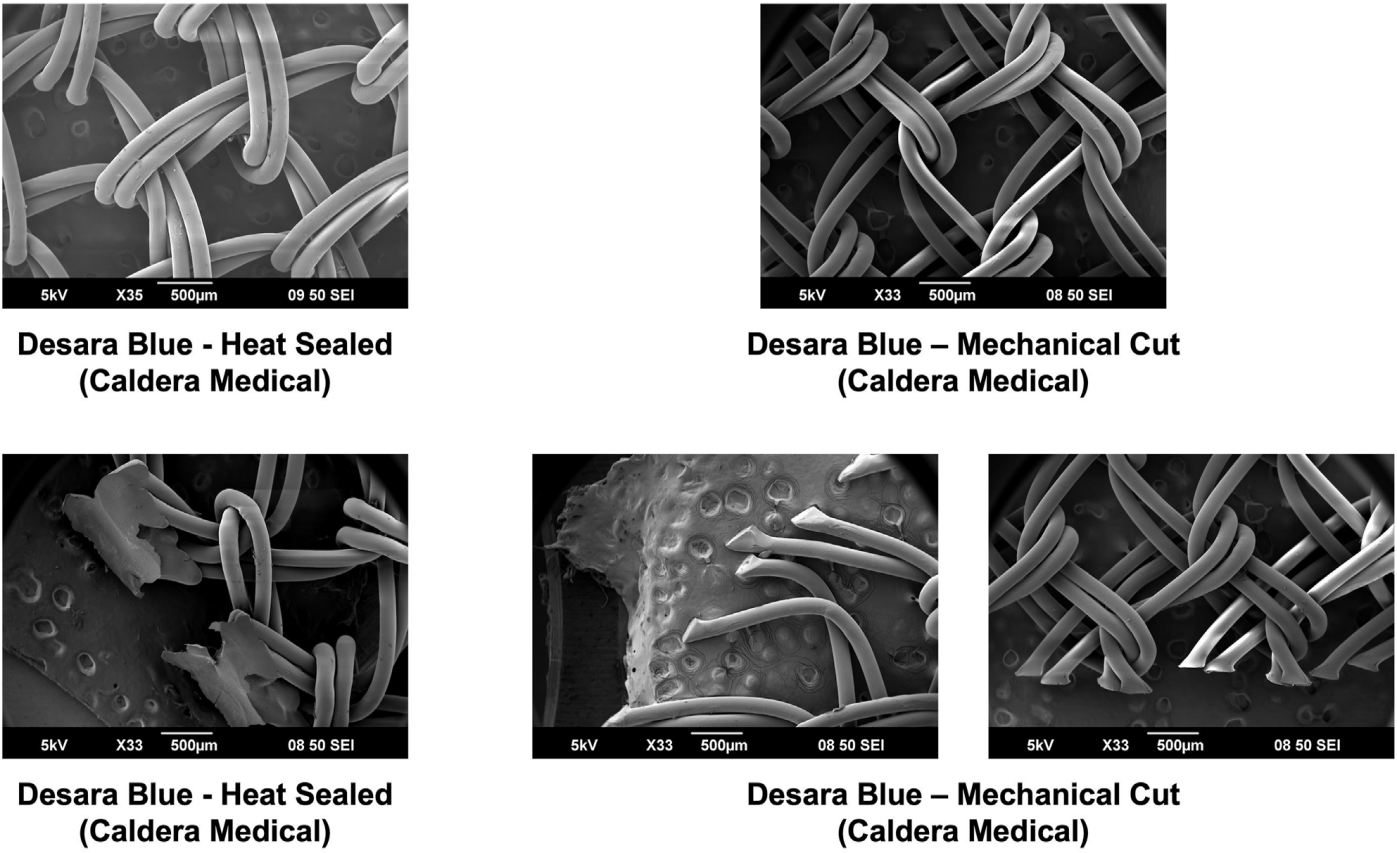
SEM images of Desara Blue heat sealed and Desara Blue mechanical cut with complete pores shown in the top images and the edges of the respective MUSs depicted in the bottom images. Similar to the other MUSs analyzed in this study, the Desara Blue MUSs have a general pore shape that is quadrilateral. The edges of Desara Blue heat sealed have a thick edge resulting from the neighboring tanged edges melting together whereas the edges of Desara Blue mechanical cut were left free with the tanged edges on one side being longer than the opposing side.

Overall, the general pore shape of the MUSs analyzed in this study was a quadrilateral (Figures 1–3). As anticipated, differences were observed along the edges of the MUSs. Desara Blue mechanical cut edges were similar to the TVT and were “tanged” meaning where a knot was disrupted during the cutting process, the edges were left free (Figures 2,3 [Fig F3]). The Desara Blue heat sealed sling, in contrast, had a thick edge formed from neighboring tanged edges melting together (Figure 3). Finally, Gynecare TVT Exact (TVT Exact) and ArcTV had similar edges that were individually sealed (laser cut), a more precision focused modification barely perceptible to the naked eye and did not coalesce together like heat sealed edges.

### 3.2 Tensile testing

**FIGURE 4:**
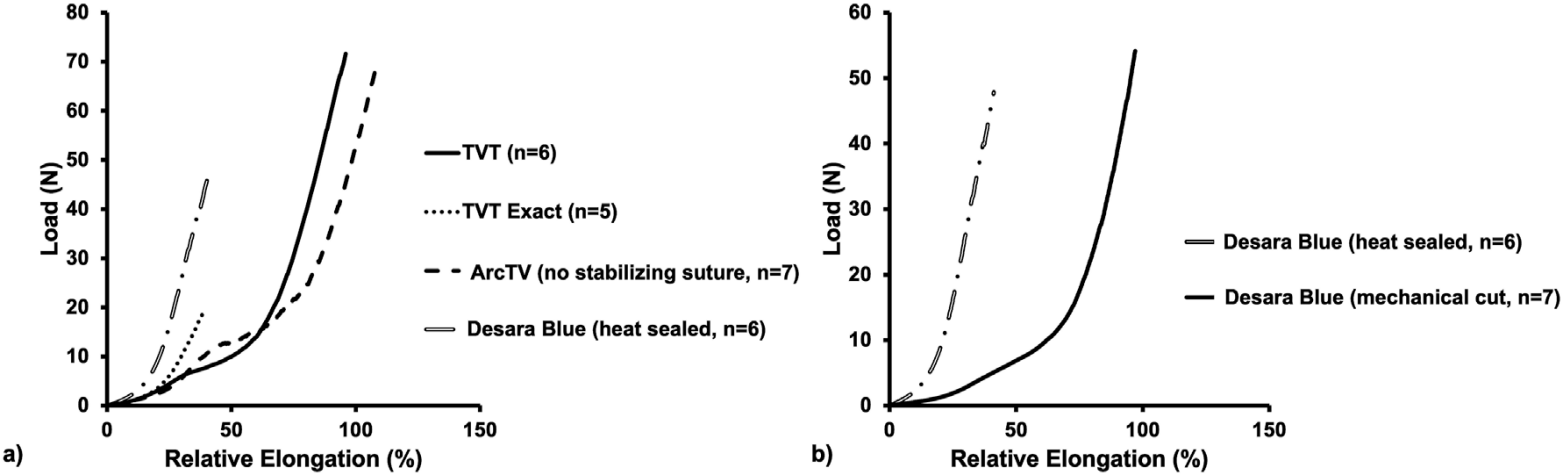
Average load-relative elongation curves following load-to-failure testing of the commercially available MUSs **(a)** and the commercially available Desara Blue (heat sealed) versus the non-commercially available Desara Blue (mechanical cut) **(b)**.

Following tensile load to failure testing, all MUSs demonstrated the typical bilinear load-relative elongation curve previously described for MUSs with an initial low stiffness region followed by a transition region (quantified by the inflection point), culminating into a high stiffness region (Figure 4). It is noteworthy, that at approximately 50% relative elongation, the load-relative elongation curves of TVT Exact exhibited premature failures which were apparent as repetitive peaks and valleys in the load-relative elongation curve (Supplementary Figure S1). These premature failures were not observed for TVT and therefore, were presumed to be failures at the laser cut edges. As shown in Supplementary Figure S1, the load-relative elongation curve for the TVT depicts a device that 1) easily deforms with loading and 2) is less stiff than TVT Exact. Upon close examination, the premature failures observed with TVT Exact were confirmed to be the result of disruption of the laser cut edges and were visualized as small fragments breaking away from the device (Supplementary Video S1). This phenomenon, however, would only occur with substantial overtensioning of the MUS and rarely occurs *in vivo* given that most of the disruptions occurred at loads that were beyond the physiologic range (and relative elongations of 1.5 to 2X their original value). For example, a sling with an original length of 8 cm is not expected to increase to 12 cm *in vivo*. Thus, the load-relative elongation curves for TVT Exact were truncated to exclude this premature failure region, which likely resulted in an underestimation of the structural properties, specifically the load at failure and elongation at failure for TVT Exact. Importantly, there were instances in which small fibers were observed breaking away from TVT, ArcTV, Desara Blue heated sealed, and Desara Blue mechanical cut MUSs when loaded beyond the physiologic range (Supplementary Videos S2–S5); however, these did not impact the load-relative elongation curves to the extent that they did for the TVT Exact. It is important to keep in mind that aside from cutting the MUSs to the appropriate length for testing, no other alterations were done to the MUSs. Additionally, all mid-urethral slings when loaded to failure elongated beyond the physiologic range (estimated to end at ∼25% relative elongation). Therefore, the structural properties were presented up to failure, and the load-relative elongation curves for all MUSs were truncated at 25% relative elongation from which the low and high stiffnesses were calculated and reported.

#### 3.2.1 Structural properties up to failure

The load-relative elongation curves up to failure were similar for TVT, TVT Exact, and ArcTV (no stabilizing suture) within the initial low stiffness region (up to ∼14% relative elongation). Beyond 14% relative elongation, the load-relative elongation curve of ArcTV most closely resembled that of TVT (Figure 4a). Quantifying the structural properties (Table 2), revealed that the low stiffness, high stiffness, and relative elongation at failure were not significantly different between TVT and ArcTV [all p-values>0.05], corroborating the observation that ArcTV responded to loading similar to the TVT. The TVT, however, was approximately 18% stronger (i.e., failed at a higher load, p = 0.001) and the relative elongation at the transition point between the low and high stiffness regions (i.e., the relative elongation at the inflection point) was roughly 15% lower than ArcTV (p = 0.001). The low stiffness of TVT Exact was not significantly different from that of TVT (p = 0.792). All other structural properties for TVT Exact were significantly less than that of TVT (all p-values = 0.004), and this is likely the result of the premature failures experienced by TVT Exact, which resulted in the truncated load-relative elongation curve.

**TABLE 2:** Structural properties obtained following a uniaxial tensile test to failure.

	Low stiffness (N/mm)	High stiffness (N/mm)	Load at failure (N)	RE at failure (%)	RE at the inflection point (%)
TVT (mechanical cut, n = 6)	0.10 ± 0.03	2.2 ± 0.3	91.0 ± 6.3	105.3 ± 10.9	65.2 ± 5.3
TVT Exact (laser cut, n = 5)	0.10 ± 0.01	1.2 ± 0.2	26.4 ± 4.7^[Table-fn Tfn3]	46.0 ± 5.7^	22.2 ± 1.9
ArcTV (laser cut, no stabilizing suture, n = 7)	0.12 ± 0.01	1.9 ± 0.2	76.8 ± 2.4	112.3 ± 3.3	76.4 ± 3.5
Desara Blue (heat sealed, n = 7)	0.23 ± 0.02	2.3 ± 0.2	61.1 ± 10.8	52.4 ± 9.3	19.6 ± 2.3
Desara Blue (mechanical cut, n = 7)^§^	0.06 ± 0.01	2.0 ± 0.2	69.1 ± 6.6	103.3 ± 3.8	70.2 ± 2.2
Overall p-value^*^	0.001^a^	<0.001^a^	<0.001^a^	<0.001^a^	<0.001^a^
TVT vs. TVT Exact	0.792^b^	0.004^b^	0.004^b^	0.004^b^	0.004^b^
TVT vs. ArcTV (no stabilizing suture)	0.234^b^	0.073^b^	0.001^b^	0.295^b^	0.001^b^
TVT vs. Desara Blue (heat sealed)	0.002^b^	0.485^b^	0.002^b^	0.002^b^	0.002^b^
Desara Blue heat sealed vs. mechanical cut	0.001^c^	0.014^c^	0.181^c^	0.001^c^	0.001^c^

RE, relative elongation.

Data represented as mean ± standard deviation.

^ TVT Exact exhibited premature failures; thus, the load-relative elongation curves for TVT Exact were truncated, which likely resulted in an underestimate of the load at failure and elongation at failure for TVT Exact.

^*^ P-value obtained using:

^a^ Kruskal-Wallis, followed by

^b^ Mann-Whitney U tests with a Bonferroni correction (adjusted α = 0.0167).

^§^ Desara Blue mechanical cut is not commercially available; therefore, Desara Blue mechanical cut was not compared to all slings. A separate analysis was performed to assess the impact of heat by comparing Desara Blue heat sealed vs Desara Blue mechanical cut (pvalue obtained using ^c^Mann-Whitney U test).

Initially, the stiffness of Desara Blue heat sealed was 130% higher than that of the TVT (p = 0.002) as quantified by the low stiffness value; however, the high stiffness was not significantly different between the two mid-urethral slings (p = 0.485). The load at failure, relative elongation at failure, and relative elongation at the inflection point were all significantly higher for TVT as compared to Desara Blue heat sealed meaning that it deformed more easily (all p-values = 0.002). Lastly, the low and high stiffness values of Desara Blue heat sealed were 283% (p = 0.001) and 15% (p = 0.014) higher than Desara Blue mechanical cut, respectively. Additionally, the relative elongation at failure and relative elongation at the inflection point were 97% (p = 0.001) and 258% (p = 0.001) higher for Desara Blue mechanical cut relative to Desara Blue heat sealed due to its higher stiffness. These results are consistent with the load-relative elongation curves in which the curve for Desara Blue mechanical cut was less steep and shifted towards the right (meaning it elongated more) as compared to Desara Blue heat sealed (Figure 4b). Overall, the behavior of Desara heat-sealed is reflective of a substantially stiffer product.

#### 3.2.2 Low stiffness and high stiffness up to 25% relative elongation

**FIGURE 5:**
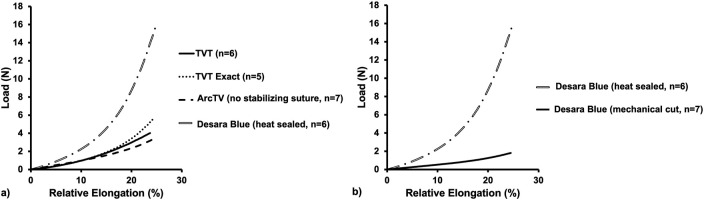
Average load-relative elongation curves up to 25% relative elongation of the commercially available MUSs **(a)** and the commercially available Desara Blue (heat sealed) versus the non-commercially available Desara Blue (mechanical cut) **(b)**.

Conducting uniaxial tensile testing to failure is a common protocol used to assess the maximum properties of a material/device (e.g., how much force or elongation the material/device will withstand prior to failing). However, the loads (via intra-abdominal pressures) experienced by mid-urethral slings with physiologic loading are not within the failure range. Thus, all load-relative elongation curves were truncated at 25% of relative elongation (Figure 5), which corresponds to approximately 15 N for the MUSs analyzed in this study. The low stiffness and high stiffness were then re-calculated as the minimum and maximum slope, respectively, over a 2% interval of relative elongation (Table 3). Qualitatively, TVT, TVT Exact, and ArcTV (no stabilizing suture) curves followed a similar path until reaching ∼12% relative elongation at which point the curves began to separate from TVT Exact which had a steeper slope (indicating a higher stiffness) followed by TVT and ArcTV. The curve for Desara Blue heat sealed, however, had the steepest slope distinguishing it as substantially stiffer than the other MUSs. A similar result was observed when comparing Desara Blue heat sealed vs. mechanical cut in that the Desara Blue heat sealed load-relative elongation curve had a steeper slope.

**TABLE 3:** Low and high stiffness up to 25% relative elongation.

	Low stiffness (N/mm)	High stiffness (N/mm)
TVT (n = 6)	0.06 ± 0.01	0.33 ± 0.02
TVT Exact (n = 5)	0.08 ± 0.01	0.69 ± 0.12
ArcTV (no stabilizing suture, n = 7)	0.10 ± 0.01	0.32 ± 0.05
Desara Blue (heat sealed, n = 6)	0.17 ± 0.02	1.91 ± 0.30
Desara Blue (mechanical cut, n = 7)^§^	0.05 ± 0.01	0.16 ± 0.02
Overall p-value^*^	<0.001^a^	<0.001^a^
TVT vs. TVT Exact	0.082^b^	0.004^b^
TVT vs. ArcTV (no stabilizing suture)	0.001^b^	0.731^b^
TVT vs. Desara Blue (heat sealed)	0.002^b^	0.002^b^
Desara Blue heat sealed vs. mechanical cut	0.001^c^	0.001^c^

Data represented as mean ± standard deviation.

^*^ P-value obtained using:

^a^ Kruskal-Wallis, followed by

^b^ Mann-Whitney U tests with a Bonferroni correction (adjusted α = 0.0167).

^§^ Desara Blue mechanical cut is not commercially available; therefore, Desara Blue mechanical cut was not compared to all slings. A separate analysis was performed to assess the impact of heat by comparing Desara Blue heat sealed vs Desara Blue mechanical cut (pvalue obtained using ^c^Mann-Whitney U test).

Quantifying the low stiffness up to 25% relative elongation which is more relevant to what occurs physiologically, there was not a significant difference between TVT and TVT Exact (p = 0.082). However, the low stiffness of ArcTV and Desara Blue heat sealed were 67% (p = 0.001) and 183% (p = 0.002) higher than TVT, respectively. Additionally, the low stiffness of the Desara Blue heat sealed sling was 240% increased (p = 0.001) as compared to Desara Blue mechanical cut. Similar trends were observed when comparing high stiffness values. The TVT Exact and Desara Blue heat sealed were 109% (p = 0.004) and 479% (p = 0.002) stiffer than the TVT high stiffness, respectively. The high stiffness of ArcTV was not different from TVT (p = 0.731). Compared to Desara Blue mechanical cut, the high stiffness of Desara Blue heat sealed was 1,094% increased (p = 0.001).

### 3.3 Permanent elongation following cyclic loading

**FIGURE 6:**
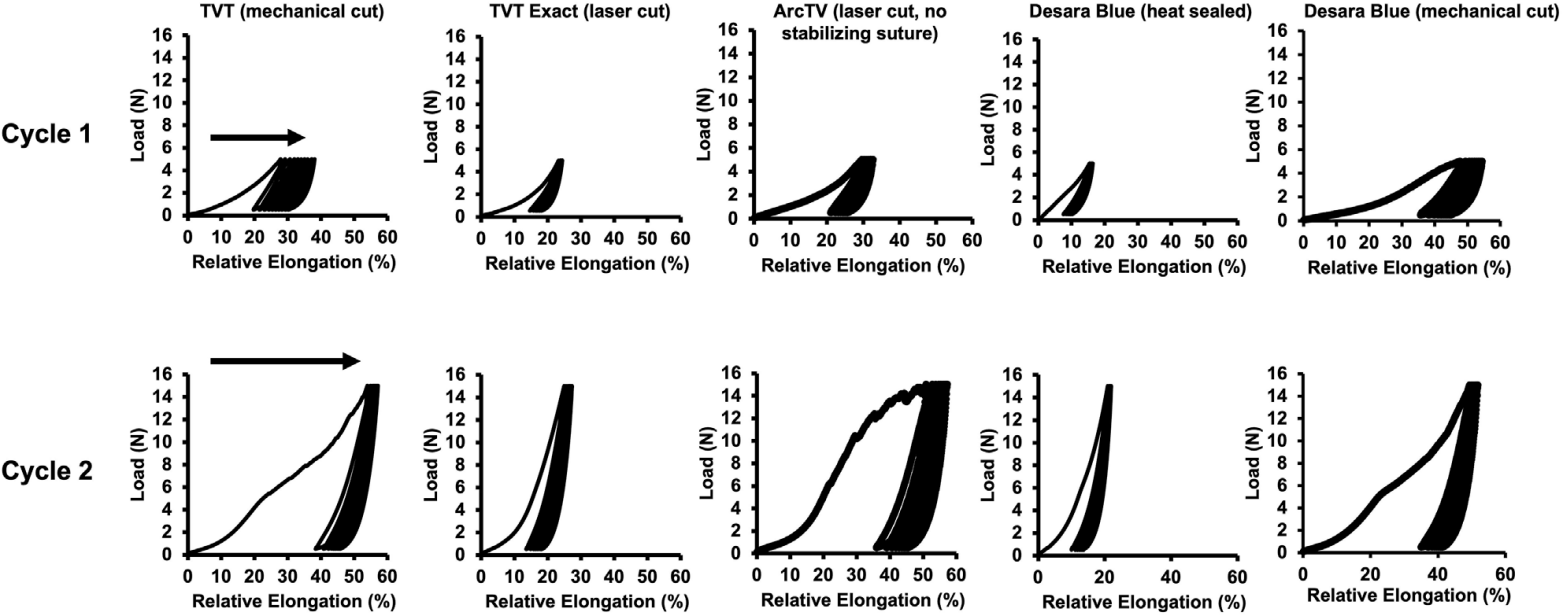
Representative load-relative elongation curves following cyclical loading after Cycle 1–0.5 N–5 N for 10 cycles and Cycle 2–0.5 N–15 N for 10 cycles. Permanent elongation is evidenced by a shift to the right (arrows) of the load-relative elongation curves.

*In vivo* it is anticipated that MUSs will undergo repetitive cycles of loading and unloading as a result of fluctuations in intra-abdominal pressure with normal physiologic functions such as sneezing, coughing, lifting, and rising from a seated position. Thus, the MUSs were subjected to cyclical loading and the amount of permanent elongation (i.e., unrecoverable lengthening of the MUSs) was quantified as a shift to the right in the peaks and valleys of the load-relative elongation curves (Figure 6). Note, ArcTV was test without the stabilizing suture for the assessment of permanent elongation.

Following Cycle 1 (0.5 N–5 N for 10 cycles), permanent elongation was significantly lower for TVT Exact (↓54%, p = 0.004) and Desara Blue heat sealed (↓75%, p = 0.001) as compared to TVT (Table 4). ArcTV (no stabilizing suture) experienced slightly less permanent elongation relative to TVT (12.1% vs. 15.5%); however, this difference was not significant (p = 0.051). Relative to Desara Blue heat sealed, permanent elongation for Desara Blue mechanical cut increased by 538% (p < 0.001). Similar results were observed following Cycle 2 (0.5 N–15 N for 10 cycles) in which TVT Exact and Desara Blue heat sealed deformed 63% (p = 0.004) and 76% (p = 0.001) less than TVT, respectively. Permanent elongation was not significantly different between TVT and ArcTV (p = 0.534). Heat sealing Desara Blue led to an 80% reduction (p < 0.001) in permanent elongation relative to mechanical cutting. The percentage of permanent elongation changed very little following Cycle 3, which was anticipated given that the protocol for Cycle 3 is the same as that for Cycle 1 (i.e., the MUSs were only loaded up to 5 N). Therefore, the same trends and significant differences following Cycle 3 were observed when comparing TVT to TVT Exact, ArcTV, and Desara Blue heat sealed and between Desara Blue heat sealed vs. Desara Blue mechanical cut.

**TABLE 4:** Permanent elongation following cyclical loading–Cycle 1 and 3 (0.5 N–5 N) and Cycle 2 (0.5 N–15 N).

	Permanent elongation after Cycle 1 (%)	Permanent elongation after Cycle 2 (%)	Permanent elongation after Cycle 3 (%)
TVT (mechanical cut, n = 6)	15.5 ± 2.8	46.2 ± 6.1	47.8 ± 5.1
TVT Exact (laser cut, n = 5)	7.1 ± 1.0	17.2 ± 1.3	17.2 ± 1.6
ArcTV (laser cut, no stabilizing suture, n = 7)	12.1 ± 2.2	43.5 ± 3.0	43.9 ± 3.0
Desara Blue (heat sealed, n = 7)	3.9 ± 0.6	11.0 ± 1.4	11.0 ± 1.8
Desara Blue (mechanical cut, n = 7)^§^	24.9 ± 4.8	55.8 ± 5.6	56.2 ± 5.2
Overall p-value^*^	<0.001^a^	<0.001^a^	<0.001^a^
TVT vs. TVT Exact	0.004^b^	0.004^b^	0.004^b^
TVT vs. ArcTV (no stabilizing suture)	0.051^b^	0.534^b^	0.101^b^
TVT vs. Desara Blue (heat sealed)	0.001^b^	0.001^b^	0.001^b^
Desara Blue heat sealed vs. mechanical cut	<0.001^c^	<0.001^c^	<0.001^c^

Data represented as mean ± standard deviation.

^*^ P-value obtained using:

^a^ Kruskal-Wallis, followed by

^b^ Mann-Whitney U tests with a Bonferroni correction (adjusted α = 0.0167).

^§^ Desara Blue mechanical cut is not commercially available; therefore, Desara Blue mechanical cut was not compared to all slings. A separate analysis was performed to assess the impact of heat by comparing Desara Blue heat sealed vs Desara Blue mechanical cut (pvalue obtained using ^c^Mann-Whitney U test).

### 3.4 Porosity quantification

**FIGURE 7:**
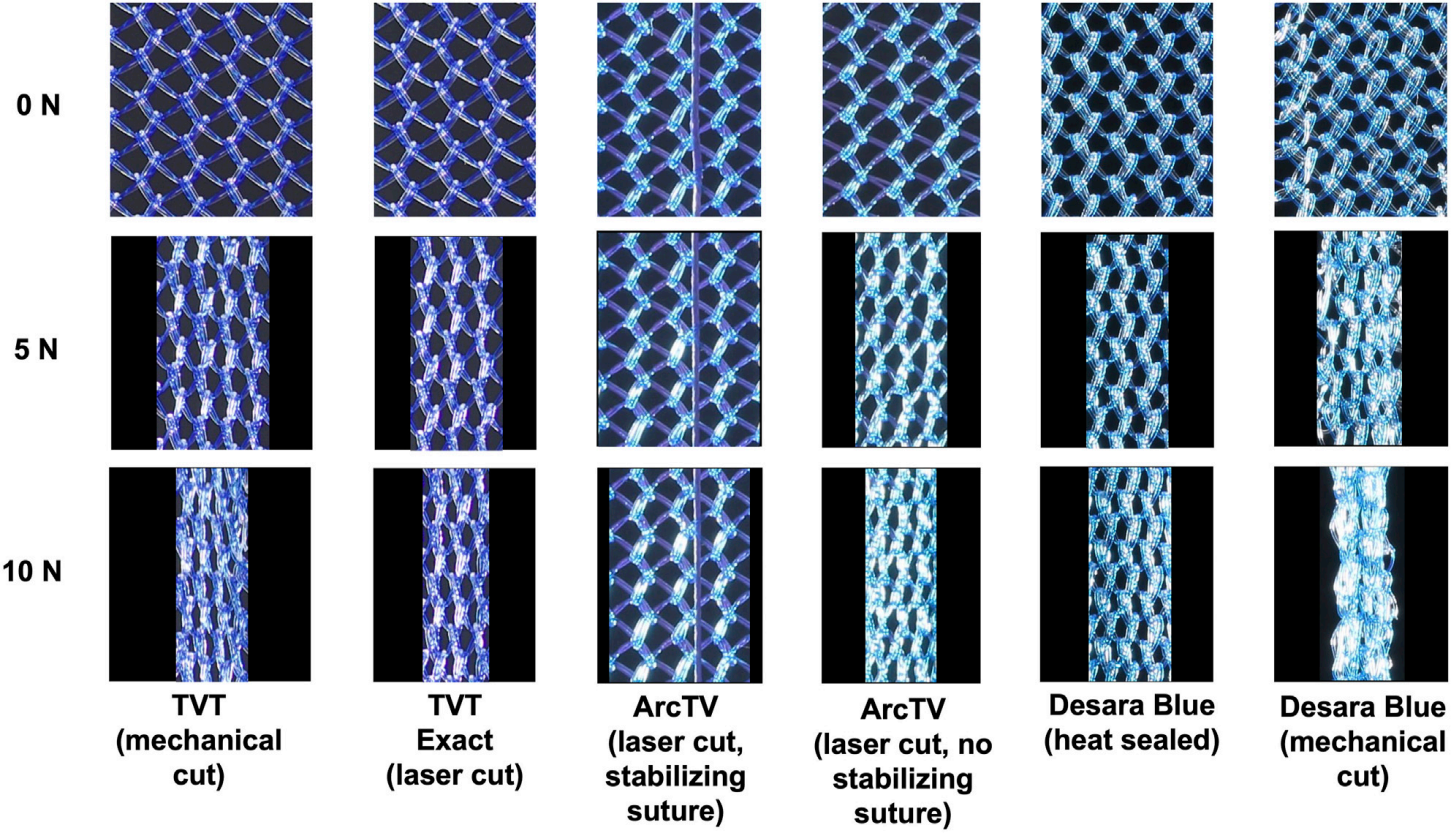
Representative images of the mid-section of the MUSs after the application of 0 N, 5 N, and 10 N. All slings contracted and the pores collapsed except ArcTV with the stabilizing suture in which the sling experienced little contraction and the pores remained open.

Prior to loading, the pores of all mid-urethral slings were open. However, with loading a dramatic alteration in pore geometry occurred for all MUSs except for ArcTV with the stabilizing suture (Figure 7). At 5 N, nearly all the mid-urethral slings contracted in the lateral direction while the pores remained relatively open and were elongated for TVT, TVT Exact, ArcTV without the stabilizing suture, and Desara Blue heat sealed. This contrasts with Desara Blue mechanical cut in which the pores were nearly all collapsed. ArcTV with the stabilizing suture was the only MUS in which the pores remained open and were geometrically unaltered by loading. Increasing the load to 10 N resulted in increased pore elongation and MUS contraction with the greatest change in pore geometry (i.e., complete pore collapse) observed for Desara Blue mechanical cut followed by TVT. Similar to the results at 5 N, the lowest change in pore geometry at 10 N was observed for ArcTV with the stabilizing suture (i.e., the pores remained relatively open with minimal MUS contraction) (Figure 7).

**FIGURE 8:**
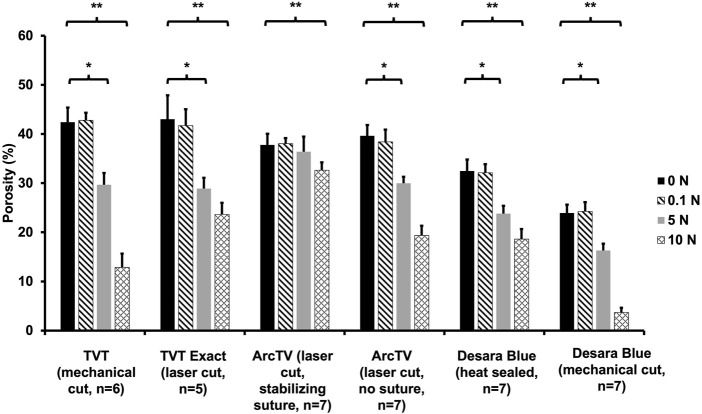
Average porosity following the application of 0 N, 0.1 N, 5 N, and 10 N. A significant difference in porosity between 0.1 N and 5 N is represented by (*) and between 0.1 N and 10 N is represented by (**). Error bars represent standard deviation.

Quantifying the porosity and pore size (average minimal and maximum pore diameter, see Supplementary Figures S2,S3, respectively) confirmed the qualitative observations (Figure 8; Table 5). Increasing the load from 0 N to 5 N resulted in a significant decline in porosity by approximately 24%–33% for all mid-urethral slings (all p-values <0.001) except for ArcTV with the stabilizing suture in which the porosity at 5 N was not different from 0 N (p = 0.739). Increasing the load to 10 N, the largest reduction in porosity was observed for Desara Blue mechanical cut which decreased by 83% (p < 0.001) followed by TVT, decreasing by 70%, p < 0.001. Overall, the smallest decline in porosity was observed for ArcTV with the stabilizing suture, reducing 14% (p < 0.001) at 10 N. Given that the starting porosity of each mid-urethral sling was different, the percent change from 0.1 N was calculated and used to compare porosity changes across all mid-urethral slings. Furthermore, since ArcTV with the stabilizing suture experienced the smallest change in porosity, the percent change in porosity for all mid-urethral slings (while controlling for the load applied) was compared to ArcTV with the stabilizing suture. Indeed, the percent change in porosity (loss of porosity represented as a negative number) for all mid-urethral slings was significantly increased with adjusted mean differences ranging from −18 to −32 (all p-values<0.001, Table 6) relative to ArcTV with the stabilizing suture. A significant difference in the percent change in porosity was not observed between TVT and TVT Exact (p = 0.201) whereas Desara Blue mechanical cut experienced a greater reduction in the percent change in porosity as compared to Desara Blue heat sealed (p < 0.001).

**TABLE 5:** Change in porosity with increasing load.

Load	Porosity (%)	Unadjusted mean difference (95% CI)	P-value^a^	Load	Porosity (%)	Unadjusted mean difference (95% CI)	P-value^a^
	Mean (SD)				Mean (SD)		
TVT (mechanical cut, n = 6)				TVT exact (laser cut, n = 5)			
0 N	42.4 (3.0)	Referent	<0.001^b^	0 N	43.0 (4.9)	Referent	<0.001^b^
0.1 N	42.8 (1.5)	0.4 (−1.10, 1.90)	0.602	0.1 N	41.7 (3.3)	−1.3 (−3.03, 0.43)	0.141
5 N	29.7 (2.4)	−12.7 (−14.6, −10.8)	<0.001	5 N	28.9 (2.2)	−14.1 (−19.4, −8.87)	<0.001
10 N	12.9 (2.8)	−29.5 (−32.5, −26.6)	<0.001	10 N	23.6 (2.3)	−19.4 (−25.3, −13.5)	<0.001
ArcTV with stabilizing suture (laser cut, n = 7)				ArcTV without stabilizing suture (laser cut, n = 7)			
0 N	37.8 (2.3)	Referent	<0.001^b^	0 N	39.6 (2.2)	Referent	<0.001^b^
0.1 N	38.0 (1.1)	0.26 (−1.25, 1.77)	0.739	0.1 N	38.3 (2.5)	−1.29 (−2.44, −0.13)	0.029
5 N	36.4 (3.1)	−1.39 (−4.35, 1.58)	0.360	5 N	30.0 (1.3)	−9.64 (−11.3, −7.97)	<0.001
10 N	32.6 (1.6)	−5.13 (−7.08, −3.17)	<0.001	10 N	19.4 (2.0)	−20.3 (−22.4, −18.1)	<0.001
Desara Blue (heat sealed, n = 7)				Desara Blue (mechanical cut, n = 7)			
0 N	32.5 (2.3)	Referent	<0.001^b^	0 N	23.9 (1.7)	Referent	<0.001^b^
0.1 N	32.1 (1.7)	−0.36 (−1.49, 0.78)	0.536	0.1 N	23.9 (1.5)	6.11e-16 (−0.43, 0.43)	1.000
5 N	23.8 (1.6)	−8.67 (−10.26, −7.09)	<0.001	5 N	17.3 (2.1)	−6.6 (−8.39, −4.81)	<0.001
10 N	18.6 (2.0)	−13.9 (−16.7, −11.08)	<0.001	10 N	4.0 (1.3)	−19.9 (−21.6, −18.3)	<0.001

^a^ P-value from mixed effects linear regression.

^b^ Overall p-value from unadjusted mixed effects linear regression model.

**TABLE 6:** Percent change in porosity relative to ArcTV with stabilizing suture.

	Percent change in porosity (%) Mean (SD)	Adjusted^a^ mean difference (95% CI)	P-value^b^
ArcTV (stabilizing suture, n = 21)	−5.2 (9.7)	Referent	
TVT (n = 18)	−32.8 (30.2)	−27.5 (−32.4, −22.7)	<0.001
TVT Exact (n = 15)	−26.2 (20.2)	−21.0 (−30.7, −11.3)	<0.001
ArcTV (no stabilizing suture, n = 21)	−26.1 (20.5)	−20.9 (−27.9, −14.0)	<0.001
Desara Blue (heat sealed, n = 21)	−23.2 (18.5)	−18.0 (−26.7, −9.28)	<0.001
Desara Blue (mechanical cut, n = 21)	−36.9 (36.0)	−31.6 (−39.2, −24.1)	<0.001
Pairwise comparisons			
TVT vs. TVT Exact		6.56 (−3.49, 16.6)	0.201^c^
Desara Blue heat sealed vs. mechanical cut		−13.7 (−17.2, −10.1)	<0.001^c^
Load			
0.1 N (n = 39)	−0.7 (4.4)	Referent	
5 N (n = 39)	−23.3 (12.3)	−22.6 (−25.1, −20.1)	<0.001
10 N (n = 39)	−50.4 (24.0)	−49.7 (−52.1, −47.3)	<0.001

^a^ Adjusted for load.

^b^ P-value from mixed effects linear regression.

^c^ P-value based on the z-statistic for the difference between coefficients estimated by the parent model.

## 4 Discussion

Implantation with a mid-urethral sling (MUS) is the gold-standard method for the treatment of stress urinary incontinence (Cox et al., 2013). The safety and efficacy of mid-urethral slings has been extensively studied with over 2000 scientific publications globally describing the treatment of SUI with MUSs, including multiple studies with follow-ups at 17-years or more (Tulokas et al., 2020; Ogah et al., 2011; Nilsson et al., 2013; Lorenzo-Gómez et al., 2024; Al-Zahrani and Gajewski, 2016; Goessens and Cammu, 2023). All mid-urethral slings are knitted, macroporous, have a common pore geometry, and are composed of monofilament polypropylene, yet manufacturers use various techniques such as laser cutting, heat sealing, and the inclusion of an interwoven stabilizing suture to reduce deformation of the MUS. This study aimed to assess how these modifications impact the mechanical behavior (i.e., deformation and change in pore geometry with loading) of mid-urethral slings by comparing the structural properties, permanent elongation, and porosity of devices that are laser cut, heat sealed, and have an interwoven stabilizing suture to the mechanical cut TVT (the prototype MUS). To assess the impact of laser cutting, the TVT (mechanical cut) was compared to the TVT Exact (laser cut) and to assess the additional impact of a stabilizing suture, the change in porosity with loading of ArcTV was evaluated with and without the stabilizing suture. Lastly, to evaluate the impact of heat sealing, Desara Blue heat sealed (a commercially available MUS) was compared to the non-commercially available MUS, Desara Blue mechanical cut.

Congruent with the study hypothesis, the application of heat to the edges via heat-sealing (laser cutting to a lesser extent) increased stiffness and reduced the elongation at failure of the MUS compared to mechanical cutting, in which the edges are left untreated. Additionally, the increased stiffness resulting from heat treatment significantly reduced the amount of permanent elongation in response to cyclical loading for the laser cut and heat-sealed MUSs. This was particularly observed when comparing Desara Blue heat-sealed versus Desara Blue mechanical cut and TVT versus TVT Exact. From a surgeon’s perspective, a heat-treated MUS would be beneficial for reducing deformation (i.e., maintaining the shape of the MUS) during handling and surgical placement. Thus, if a surgeon were to apply a small load (<5 N) during implantation, a high stiffness/heat treated MUS would be less likely to elongate or permanently change shape relative to a mechanical cut MUS. Similarly, *in vivo* a high stiffness/heat treated MUS would be less likely to elongate in response to small sudden changes in load (e.g., a small cough or sneeze) or to small consecutive changes in load (e.g., small, repeated coughs or sneezes) relative to a mechanical cut MUS. However, it is important to keep in mind that *in vivo* it is anticipated that tissue will grow within the pores of the MUS thus impacting how much the MUS deforms *in vivo*. Future studies are needed to assess how the incorporation of tissue within the MUS will impact deformation. Nevertheless, the cost of increasing stiffness, however, is the substantial stiffness mismatch with the urethra making the MUS more likely to cause tissue damage due to micro-motion (i.e., a stiffer material, MUS, moving against a softer tissue) and stress shielding whereby the stiff MUS would shield the tissue from experiencing the normal physiologic loads thus causing the tissue to atrophy (Rumian et al., 2009; Yamamoto et al., 1993). This would be particularly true of younger reproductive aged women whose tissues are softer than those of older women. Previously, Shapiro et al. found evidence of stress shielding *in vivo* with increased stiffness of a single incision sling in an ovine model (Shapiro et al., 2020). Studies on pelvic organ prolapse (prolapse) meshes implanted in a nonhuman primate model have also demonstrated 1) vaginal degeneration and atrophy (i.e., stress shielding), particularly with the implantation of a high stiffness mesh (Feola et al., 2013; Jallah et al., 2016; Liang et al., 2013; Shaffer et al., 2019), and 2) evidence of injury due to micromotion (Artsen et al., 2019; Tennyson et al., 2019). In an *ex vivo* study using porcine abdominal muscle, Schmidt et al. found that erosion rates were higher for heat treated MUSs versus mechanical cut MUSs (Schmidt and Taylor, 2021). Based on these studies, it is hypothesized that MUSs, particularly those that are heat treated, will have a similar impact on the surrounding tissue as mini-slings and prolapse meshes and would not be recommended for use by surgeons; however, future studies are needed to test this hypothesis using an *in vivo* approach.

In addition to impacting the structural properties and percent of permanent elongation, the way that the edges of MUSs are finished also influences the tendency of small fibers to break away (referred to as fly aways) from the MUSs during loading. Specifically, MUSs that were cut closer to knots (i.e., intersecting fibers) were more prone to disruptions (aka “fly aways”) and this occurred primarily with laser cut edges, particularly with the TVT Exact. Fly aways were also but less often observed for the mechanical cut MUSs given that the edges tended to unravel before fly aways were observed. Similarly, little to no fly aways were observed with Desara Blue heat sealed, suggesting that heat sealing the edges may serve as a technique for preventing fly aways; however, the increased amount of material along the edges of Desara Blue heat sealed is likely to increase the host response to the MUS. Overall, small fibers breaking away from the MUSs is detrimental as it 1) compromises the structural integrity of the MUS and 2) studies have demonstrated that particles of different sizes can induce a varied immune response (Chikaura et al., 2016; Lebre et al., 2017). However, it is important to keep in mind that fly aways were mainly observed in this study at loads and elongations that are beyond the physiologic range, yet it is imperative that manufacturers consider the potential consequences of mechanical cutting versus laser cutting versus heat sealing on the fragility of the edges of the MUS.

Incorporating an interwoven absorbable suture within the mid-urethral sling was highly effective in maintaining the integrity (i.e., pore shape and overall architecture) of the MUS. Indeed, the pores of the ArcTV mid-urethral sling with the stabilizing suture remained open, resulting in a minimal change in porosity with increased loading. In contrast, removing the stabilizing suture from ArcTV caused the pores to collapse with loading and the porosity to significantly decrease similar to the other MUSs evaluated in this study. From a surgeon’s perspective, the inclusion of a stabilizing suture would be highly beneficial as it would reduce distortion of the MUS during handling and surgical implantation. Maintaining open pores in the early stages following implantation is critical, as this will allow for tissue integration into the pores of the MUS. Indeed, previous studies in the hernia literature have demonstrated that large pore, high porosity synthetic meshes are associated with 1) improved tissue integration, 2) decreased inflammation and fibrosis, and 3) decreased risk of the foreign body responses overlapping between neighboring fibers (i.e., bridging fibrosis) relative to small pore, low porosity synthetic meshes (Greca et al., 2001; Greca et al., 2008; Klinge et al., 2002; Orenstein et al., 2012). Implanting synthetic meshes onto the vagina of nonhuman primates with the pores collapsed and the mesh wrinkled led to exposure of the mesh fibers through the vaginal epithelium and smoothing to a complete loss of the vaginal rugae as seen clinically (Knight et al., 2022). Collectively these studies demonstrate the importance of maintaining pore size and porosity for improving outcomes; however, unlike synthetic meshes used in hernia and prolapse applications, MUSs are smaller and have a lower mesh burden. Thus, *in vivo* studies are needed to assess the impact of pore size and porosity on MUS outcomes.

The methods utilized in this study to assess the structural properties and permanent elongation of the MUSs were the same as those used previously by Moalli et al., 2008, in which the TVT was also assessed (Moalli et al., 2008). The shape of the load-relative elongation curve, structural properties, and percent of permanent elongation after each cycle for the TVT in the current study mirrored those identified in that study, indicating that a direct comparison of the structural properties and permanent elongation for all MUSs assessed by Moalli et al., 2008 can be directly compared to those assessed in the current study (and to others in which the same methods are utilized). Unlike the previous study, the low and high stiffness was reported up to 25% relative elongation in the current study. Truncating the data at 25% relative elongation is beneficial as it allows for the interpretation/comparison of the behavior of the MUSs within the physiologic range, particularly when assessing the load-relative elongation curves in which the point that the curves begin to separate (the MUSs start to resist deformation more, i.e., stiffen) can be identified. This would allow surgeons to have a better understanding of how the MUS may respond *in vivo*.

The hypotheses in this study were addressed using *ex vivo* mechanical testing of MUSs in the absence of tissue integrated within the pores of the MUS. Although the results may reflect the properties and behavior of the MUS in the early period immediately following mesh implantation (i.e., before tissue incorporation), ideally tissue will eventually integrate within the pores of the MUS and this will alter the behavior of the MUS. Thus, *in vivo* data (whether from animal studies and/or clinical trials) is needed to elucidate how manufacturing techniques such as laser cutting, heat-sealing, mechanical cutting, and the inclusion of a stabilizing suture impact the host response and ultimately clinical outcomes. Such data will allow surgeons to make an informed decision when selecting the appropriate MUS for their patients. It will also aid in the design of the next-generation of products used for SUI repairs.

The primary objectives of this study were 1) to assess how mechanical cutting, laser cutting, and heat-sealing impact the structural properties and permanent elongation of MUSs and 2) to define the impact of incorporating an interwoven stabilizing suture on the porosity of a MUS. To accomplish these objectives, a limited number of MUSs were analyzed. This was done to control for the number of parameters (e.g., pore size, pore shape, thickness, knit pattern) that could also impact the structural properties and porosity. It is important to note that companies typically do not report exactly how they manufacture their respective MUS and limited data was available regarding methods of edge modification or the textile properties of TVT Exact and Desara Blue mechanical cut. Since the respective meshes by each company were the same except for the property of the edges, we were able to assume that the textile properties (e.g., thickness, pore size, and weight) and knit pattern of TVT vs. TVT Exact and Desara Blue heat sealed vs. Desara Blue mechanical cut were the same and that the observed differences were due to the treatment of the edges which may differ methodologically by manufacturer. Additionally, the MUSs assessed in this study are not exhaustive of the MUSs that are currently available on the market. It would be interesting to see how a knotless MUS (e.g., KIM^®^ System, Neomedic International, Terrassa, Spain) would influence the structural properties and porosity with loading using the same mechanical tests performed in this study.

Overall, the structural properties and percent of permanent elongation of ArcTV (without the stabilizing suture) most closely resembled that of the TVT. This is an interesting finding given that ArcTV is laser cut versus the TVT which is mechanically cut suggesting that other factors are impacting the mechanical behavior of the device such as the knit pattern and textile properties. Incorporating the interwoven stabilizing suture provided an added benefit of maintained pore geometry with loading, which will likely translate clinically to reduced distortion of ArcTV during handling and surgical placement. Furthermore, the results of this study demonstrate that laser cutting and heat sealing reduces the deformability of a mid-urethral sling by increasing the stiffness of the device. Increasing stiffness in turn decreases the compliance of the mid-urethral sling, which will potentially increase micro-motion and lead to stress shielding, two scenarios that negatively impact outcomes. Therefore, there must be a balance between stiffness and the deformability of mid-urethral slings. In practical terms, when weighing the risks, the substantial increase in stiffness incurred by heat sealing likely exceeds benefits and laser cutting is a more acceptable edge modification for reducing deformation with less impact on stiffness as compared to heat sealing. The results of this study also corroborate the incorporation of a stabilizing suture as a technique for maintaining the integrity of the pores; thus, reducing MUS distortion during handling and surgical placement. These factors are, in theory, critical when optimizing a device for maximal tissue integration. Future studies are warranted to assess the impact of manufacturing technique on tissue integration and the host response.

## Data Availability

The original contributions presented in the study are included in the article/Supplementary Material, further inquiries can be directed to the corresponding author.

## References

[B1] Abouassaly R. Steinberg J. R. Lemieux M. Marois C. Gilchrist L. I. Bourque J.-L. (2004). Complications of tension-free vaginal tape surgery: a multi-institutional review. BJU Int. 94 (1), 110–113. 10.1111/j.1464-410x.2004.04910.x 15217442

[B2] Abufaraj M. Xu T. Cao C. Siyam A. Isleem U. Massad A. (2021). Prevalence and trends in urinary incontinence among women in the United States, 2005-2018. Am. J. Obstet. Gynecol. 225 (2), 166.e1–.e12. 10.1016/j.ajog.2021.03.016 33727114

[B3] Alhamoud M. A. S. Julaih F. A. Al-Aqil H. D. H. Almalki N. A. S. Alharthi F. A. G. Alghamdi A. A. (2024). The prevalence and risk factors of stress urinary incontinence among women in Saudi Arabia: a systematic review and meta-analysis. Healthcare 12 (23), 2440. 10.3390/healthcare12232440 39685062 PMC11640814

[B4] Al-Zahrani A. A. Gajewski J. (2016). Long-term patient satisfaction after retropubic and transobturator mid-urethral slings for female stress urinary incontinence. J. Obstet. Gynaecol. Res. 42 (9), 1180–1185. 10.1111/jog.13035 27279335

[B5] Artsen A. M. Rytel M. Liang R. King G. E. Meyn L. Abramowitch S. D. (2019). Mesh induced fibrosis: the protective role of T regulatory cells. Acta biomater. 96, 203–210. 10.1016/j.actbio.2019.07.031 31326666 PMC6717663

[B6] Barone W. R. Moalli P. A. Abramowitch S. D. (2016). Textile properties of synthetic prolapse mesh in response to uniaxial loading. Am. J. Obstetrics Gynecol. 215 (3), 326.e1–326.e9. 10.1016/j.ajog.2016.03.023 PMC5161096 27001219

[B7] Bertuit J. Nzinga A.-M. L. Feipel V. (2025). Female urinary incontinence in Africa: prevalence estimates from a systematic review and meta-analysis. Int. Urogynecology J. 10.1007/s00192-025-06146-6 PMC12618370 40447864

[B8] Carter E. Johnson E. E. Still M. Al-Assaf A. S. Bryant A. Aluko P. (2023). Single‐incision sling operations for urinary incontinence in women. Cochrane Database Syst. Rev. 2023 (10). 10.1002/14651858.cd008709.pub4 PMC10604512 37888839

[B9] Chikaura H. Nakashima Y. Fujiwara Y. Komohara Y. Takeya M. Nakanishi Y. (2016). Effect of particle size on biological response by human monocyte-derived macrophages. Biosurface Biotribology 2 (1), 18–25. 10.1016/j.bsbt.2016.02.003

[B10] Cox A. Herschorn S. Lee L. (2013). Surgical management of female SUI: is there a gold standard? Nat. Rev. Urol. 10 (2), 78–89. 10.1038/nrurol.2012.243 23318365

[B11] Dejene S. Z. Funk M. J. Pate V. Wu J. M. (2022). Long-term outcomes after midurethral mesh sling surgery for stress urinary incontinence. Female pelvic Med. Reconstr. Surg. 28 (4), 188–193. 10.1097/spv.0000000000001094 34608036 PMC9169553

[B12] Diokno A. C. Brown M. B. Herzog A. R. (1991). Cough transmission pressure to the bladder and urethra among continent and incontinent elderly women. Geriatric Nephrol. Urology 1 (1), 21–28. 10.1007/bf00451858

[B13] Feng Y. He G. Fu S. Hu Y. Wang Q. Zhai L. (2025). Three-dimensional measurement and analysis of the compressor urethrae and urethra in postpartum women. Transl. Androl. Urol. 14 (4), 1036–1048. 10.21037/tau-2024-695 40376530 PMC12076226

[B14] Feola A. Abramowitch S. Jallah Z. Stein S. Barone W. Palcsey S. (2013). Deterioration in biomechanical properties of the vagina following implantation of a high-stiffness prolapse mesh. BJOG An Int. J. Obstetrics Gynaecol. 120 (2), 224–232. 10.1111/1471-0528.12077 PMC3530836 23240801

[B15] Ford A. A. Rogerson L. Cody J. D. Aluko P. Ogah J. A. (2017). Mid‐urethral sling operations for stress urinary incontinence in women. Cochrane Database Syst. Rev. 2017 (7), CD006375. 10.1002/14651858.cd006375.pub4 PMC6483329 28756647

[B16] Goessens E. M. V. Cammu H. (2023). A 10- to 20-year follow-up after tension-free vaginal tape for stress urinary incontinence. Int. Urogynecol J. 34 (9), 2107–2114. 10.1007/s00192-023-05510-8 37000213

[B17] Greca F. H. De Paula J. B. Biondo-Simões M. L. P. Da Costa F. D. Da Silva A. P. G. Time S. (2001). The influence of differing pore sizes on the biocompatibility of two polypropylene meshes in the repair of abdominal defects: experimental study in dogs. Hernia 5 (2), 59–64. 10.1007/s100290100001 11505649

[B18] Greca F. H. Souza-Filho Z. A. Giovanini A. Rubin M. R. Kuenzer R. F. Reese F. B. (2008). The influence of porosity on the integration histology of two polypropylene meshes for the treatment of abdominal wall defects in dogs. Hernia 12 (1), 45–49. 10.1007/s10029-007-0276-6 17823771

[B19] Gurol-Urganci I. Geary R. S. Mamza J. B. Duckett J. El-Hamamsy D. Dolan L. (2018). Long-term rate of mesh sling removal following midurethral mesh sling insertion among women with stress urinary incontinence. Jama 320 (16), 1659–1669. 10.1001/jama.2018.14997 30357298 PMC6233805

[B20] Howard D. Miller J. M. Delancey J. O. Ashton-Miller J. A. (2000). Differential effects of cough, valsalva, and continence status on vesical neck movement. Obstet. Gynecol. 95 (4), 535–540. 10.1097/00006250-200004000-00011 10725485 PMC1226414

[B21] Hunskaar S. Lose G. Sykes D. Voss S. (2004). The prevalence of urinary incontinence in women in four European countries. BJU Int. 93 (3), 324–330. 10.1111/j.1464-410x.2003.04609.x 14764130

[B22] Jallah Z. Liang R. Feola A. Barone W. Palcsey S. Abramowitch S. D. (2016). The impact of prolapse mesh on vaginal smooth muscle structure and function. BJOG Int. J. Obstetrics Gynaecol. 123 (7), 1076–1085. 10.1111/1471-0528.13514 PMC5201168 26301457

[B23] Jonsson F. M. Levin P. J. Wu J. M. (2012). Trends in the surgical management of stress urinary incontinence. Obstet. Gynecol. 119 (4), 845–851. 10.1097/AOG.0b013e31824b2e3e 22433349 PMC3310349

[B24] Kapoor D. S. Housami F. White P. Swithinbank L. Drake M. (2012). Maximum urethral closure pressure in women: normative data and evaluation as a diagnostic test. Int. Urogynecol J. 23 (11), 1613–1618. 10.1007/s00192-012-1770-7 22584920

[B25] Klinge U. Klosterhalfen B. Birkenhauer V. Junge K. Conze J. Schumpelick V. (2002). Impact of polymer pore size on the interface scar formation in a rat model. J. Surg. Res. 103 (2), 208–214. 10.1006/jsre.2002.6358 11922736

[B26] Knight K. M. King G. E. Palcsey S. L. Suda A. Liang R. Moalli P. A. (2022). Mesh deformation: a mechanism underlying polypropylene prolapse mesh complications *in vivo* . Acta biomater. 148, 323–335. 10.1016/j.actbio.2022.05.051 35671876 PMC9453339

[B27] Lebre F. Sridharan R. Sawkins M. J. Kelly D. J. O’Brien F. J. Lavelle E. C. (2017). The shape and size of hydroxyapatite particles dictate inflammatory responses following implantation. Sci. Rep. 7 (1), 2922. 10.1038/s41598-017-03086-0 28592868 PMC5462791

[B28] Li L. Li G. Dai S. Lu M. Peng G. Zhou Q. (2024). Prevalence and spatial distribution characteristics of female stress urinary incontinence in mainland China. Eur. Urology Open Sci. 68, 48–60. 10.1016/j.euros.2024.08.007 PMC11414689 39308641

[B29] Liang R. Abramowitch S. Knight K. Palcsey S. Nolfi A. Feola A. (2013). Vaginal degeneration following implantation of synthetic mesh with increased stiffness. BJOG An Int. J. Obstetrics Gynaecol. 120 (2), 233–243. 10.1111/1471-0528.12085 PMC3531826 23240802

[B30] Lorenzo-Gómez M. F. Flores-Carvajal J. A. Márquez-Sánchez M. T. Márquez-Sánchez G. A. Flores-Fraile J. Alves-Rodrigues F. M. (2024). Efficacy of the sub-urethral transobturator KIM system(®) for female urinary incontinence: long term results. J. Clin. Med. 13 (19), 5728. 10.3390/jcm13195728 39407787 PMC11476618

[B31] Luber K. M. (2004). The definition, prevalence, and risk factors for stress urinary incontinence. Rev. Urol. 6 Suppl 3 (Suppl. 3), S3–S9. PMC1472862 16985863

[B32] Luo J. Betschart C. Ashton-Miller J. A. DeLancey J. O. (2016). Quantitative analyses of variability in normal vaginal shape and dimension on MR images. Int. Urogynecol J. 27 (7), 1087–1095. 10.1007/s00192-016-2949-0 26811115 PMC4916004

[B33] Meng L. F. Wang M. Zhang W. Liu X. D. Zhang Y. G. (2020). Feasibility of measuring urethral pressure during female midurethral slings: case report. Med. Baltim. 99 (28), e21100. 10.1097/md.0000000000021100 PMC7360259 32664130

[B34] Moalli P. A. Papas N. Menefee S. Albo M. Meyn L. Abramowitch S. D. (2008). Tensile properties of five commonly used mid-urethral slings relative to the TVT™. Int. Urogynecology J. Pelvic Floor Dysfunct. 19(5), 655–663. 10.1007/s00192-007-0499-1 18183344

[B35] Nilsson C. G. Palva K. Aarnio R. Morcos E. Falconer C. (2013). Seventeen years' follow-up of the tension-free vaginal tape procedure for female stress urinary incontinence. Int. Urogynecol J. 24 (8), 1265–1269. 10.1007/s00192-013-2090-2 23563892

[B36] Ogah J. Cody D. J. Rogerson L. (2011). Minimally invasive synthetic suburethral sling operations for stress urinary incontinence in women: a short version cochrane review. Neurourol. Urodyn. 30 (3), 284–291. 10.1002/nau.20980 21412819

[B37] Orenstein S. B. Saberski E. R. Kreutzer D. L. Novitsky Y. W. (2012). Comparative analysis of histopathologic effects of synthetic meshes based on material, weight, and pore size in mice. J. Surg. Res. 176 (2), 423–429. 10.1016/j.jss.2011.09.031 22099590

[B38] Pace N. Artsen A. Baranski L. Palcsey S. Durst R. Meyn L. (2021). Symptomatic improvement after mesh removal: a prospective longitudinal study of women with urogynaecological mesh complications. BJOG Int. J. obstetrics Gynaecol. 128 (12), 2034–2043. 10.1111/1471-0528.16778 PMC8497415 34047446

[B39] Rumian A. P. Draper E. R. Wallace A. L. Goodship A. E. (2009). The influence of the mechanical environment on remodelling of the patellar tendon. J. bone Jt. Surg. Br. 91 (4), 557–564. 10.1302/0301-620x.91b4.21580 19336822

[B40] Sawaqed F. Al Kharabsheh A. Tout M. Zaidan M. Khashram H. AlShunaigat N. (2020). Prevalence of stress urinary incontinence and its impact on quality of life among women in Jordan: a correlational study. J. Int. Med. Res. 48 (5), 300060520925651. 10.1177/0300060520925651 32459115 PMC7273785

[B41] Schmidt A. Taylor D. (2021). Erosion of soft tissue by polypropylene mesh products. J. Mech. Behav. Biomed. Mater. 115, 104281. 10.1016/j.jmbbm.2020.104281 33348215

[B42] Shaffer R. M. Liang R. Knight K. Carter-Brooks C. M. Abramowitch S. Moalli P. A. (2019). Impact of polypropylene prolapse mesh on vaginal smooth muscle in rhesus macaque. Am. J. Obstetrics Gynecol. 221 (4), 330.e1–330.e9. 10.1016/j.ajog.2019.05.008 PMC6863072 31102587

[B43] Shapiro K. K. Knight K. M. Liang R. Cook J. King G. E. Abramowitch S. D. (2020). Comparison of 2 single incision slings on the vagina in an ovine model. Am. J. Obstetrics Gynecol. 224, 78.e1–78.e7. 10.1016/j.ajog.2020.07.005 32707267

[B44] Tähtinen R. M. Cartwright R. Tsui J. F. Aaltonen R. L. Aoki Y. Cárdenas J. L. (2016). Long-term impact of mode of delivery on stress urinary incontinence and urgency urinary incontinence: a systematic review and meta-analysis. Eur. Urol. 70 (1), 148–158. 10.1016/j.eururo.2016.01.037 26874810 PMC5009182

[B45] Tennyson L. Rytel M. Palcsey S. Meyn L. Liang R. Moalli P. (2019). Characterization of the T-cell response to polypropylene mesh in women with complications. Am. J. Obstetrics Gynecol. 220 (2), 187.e1–187.e8. 10.1016/j.ajog.2018.11.121 PMC6557122 30419195

[B46] Townsend M. K. Curhan G. C. Resnick N. M. Grodstein F. (2010). The incidence of urinary incontinence across Asian, Black, and white women in the United States. Am. J. Obstet. Gynecol. 202 (4), 378.e1–7. 10.1016/j.ajog.2009.11.021 PMC2847676 20042169

[B47] Tulokas S. Rahkola-Soisalo P. Gissler M. Mikkola T. S. Mentula M. J. (2020). Long-term re-procedure rate after mid-urethral slings for stress urinary incontinence. Int. Urogynecol J. 31 (4), 727–735. 10.1007/s00192-019-04223-1 31956938 PMC7170977

[B48] Ulmsten U. Henriksson L. Johnson P. Varhos G. (1996). An ambulatory surgical procedure under local anesthesia for treatment of female urinary incontinence. Int. Urogynecology J. Pelvic Floor Dysfunct. 7 (2), 81–86. 10.1007/bf01902378 8798092

[B49] Wu J. M. (2021). Stress incontinence in women. N. Engl. J. Med. 384 (25), 2428–2436. 10.1056/nejmcp1914037 34161707

[B50] Yamamoto N. Ohno K. Hayashi K. Kuriyama H. Yasuda K. Kaneda K. (1993). Effects of stress shielding on the mechanical properties of rabbit patellar tendon. J. Biomech. Eng. 115 (1), 23–28. 10.1115/1.2895466 8445894

