# Supplementary material for: Characterizing the structural properties and porosity of mid-urethral slings with varied manufacturing techniques
Source: Front Bioeng Biotechnol. 2025 Aug 7;13:1543808. doi: 10.3389/fbioe.2025.1543808 (PMC12368780; doi:10.3389/fbioe.2025.1543808)
Supplement: Supplementary file 6 [file DataSheet1.docx]

Supplementary Material

**Characterizing the Structural Properties and Porosity of Mid-Urethral Slings with Varied Manufacturing Techniques**

Katrina Knight*, Leslie Meyn, Pamela Moalli

*** Correspondence:** Katrina Knight: kmk144@pitt.edu

# Supplementary Figures

**
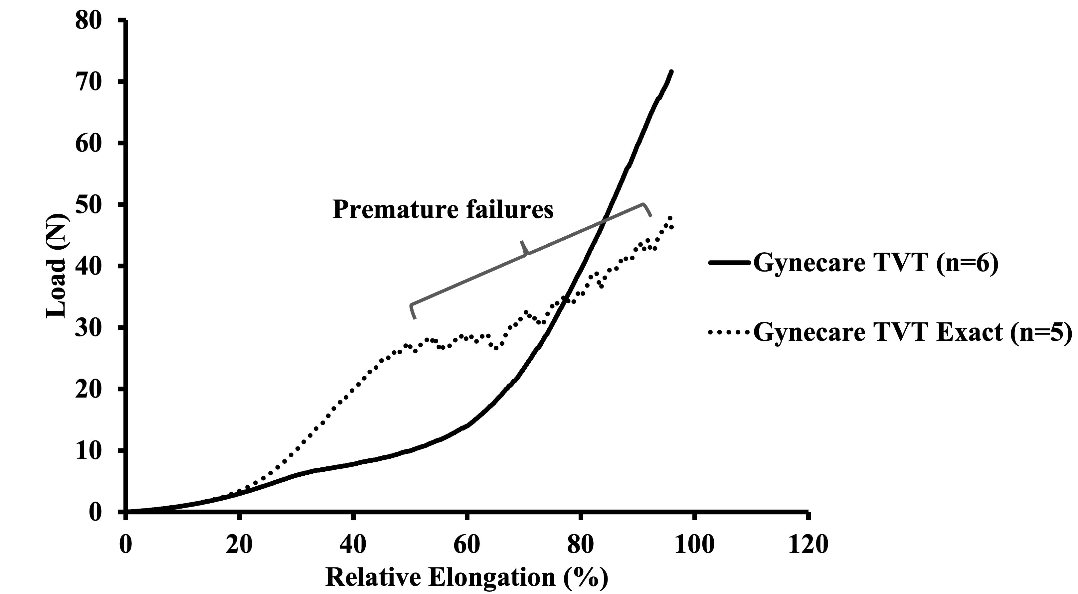
**

**Supplementary Material Figure 1.** Average load-relative elongation curves for TVT and TVT Exact demonstrating premature failures that occurred at around 50% of relative elongation for TVT Exact. The observed premature failures would likely not occur in vivo; therefore, this region was excluded from the quantification of the structural properties.

**
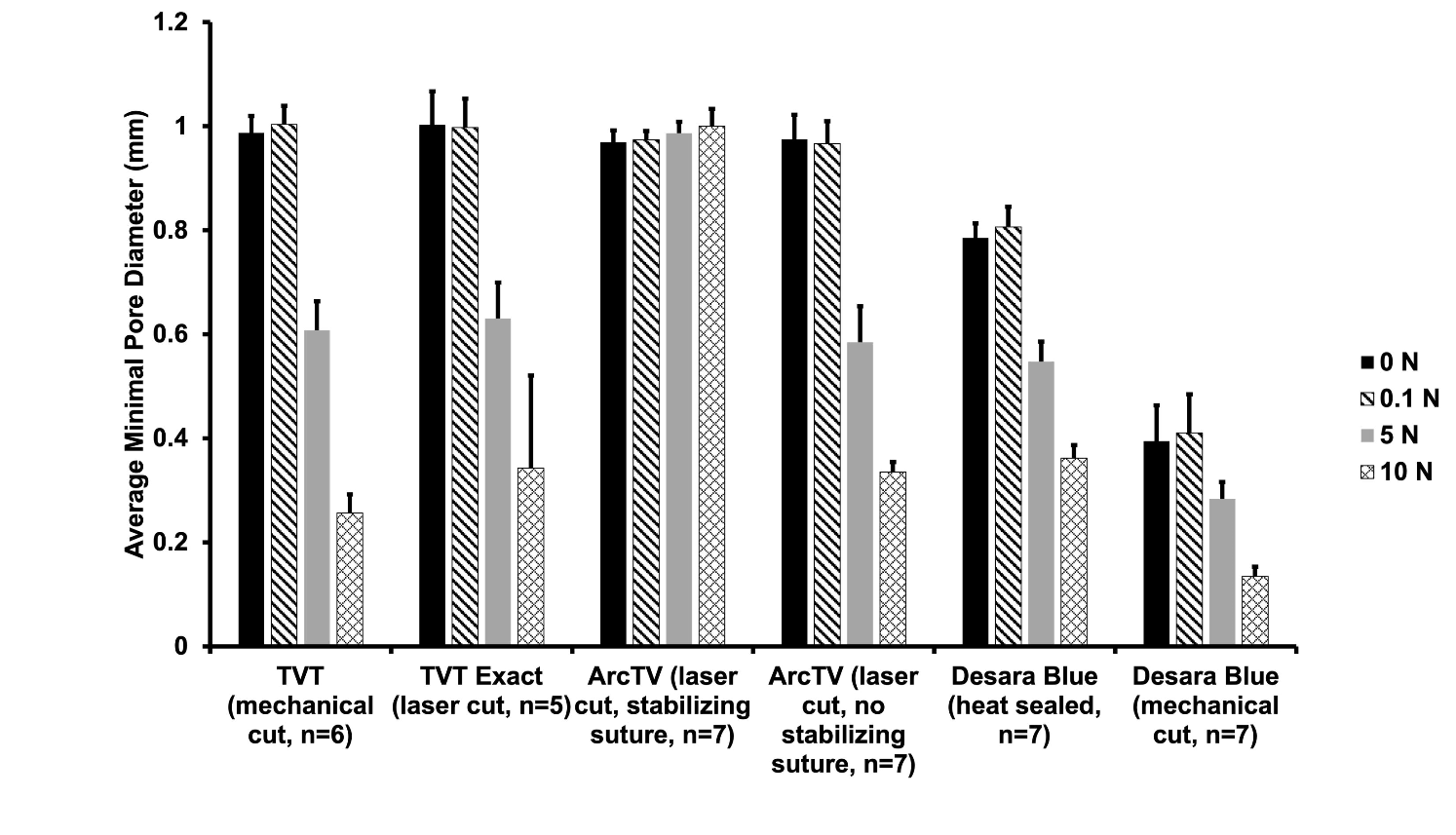
**

**Supplementary Material Figure 2.** Average minimal pore diameter following the application of 0 N, 0.1 N, 5 N, and 10 N. Overall, the minimal pore diameter decreases with increasing load. Error bars represent standard deviation.

**
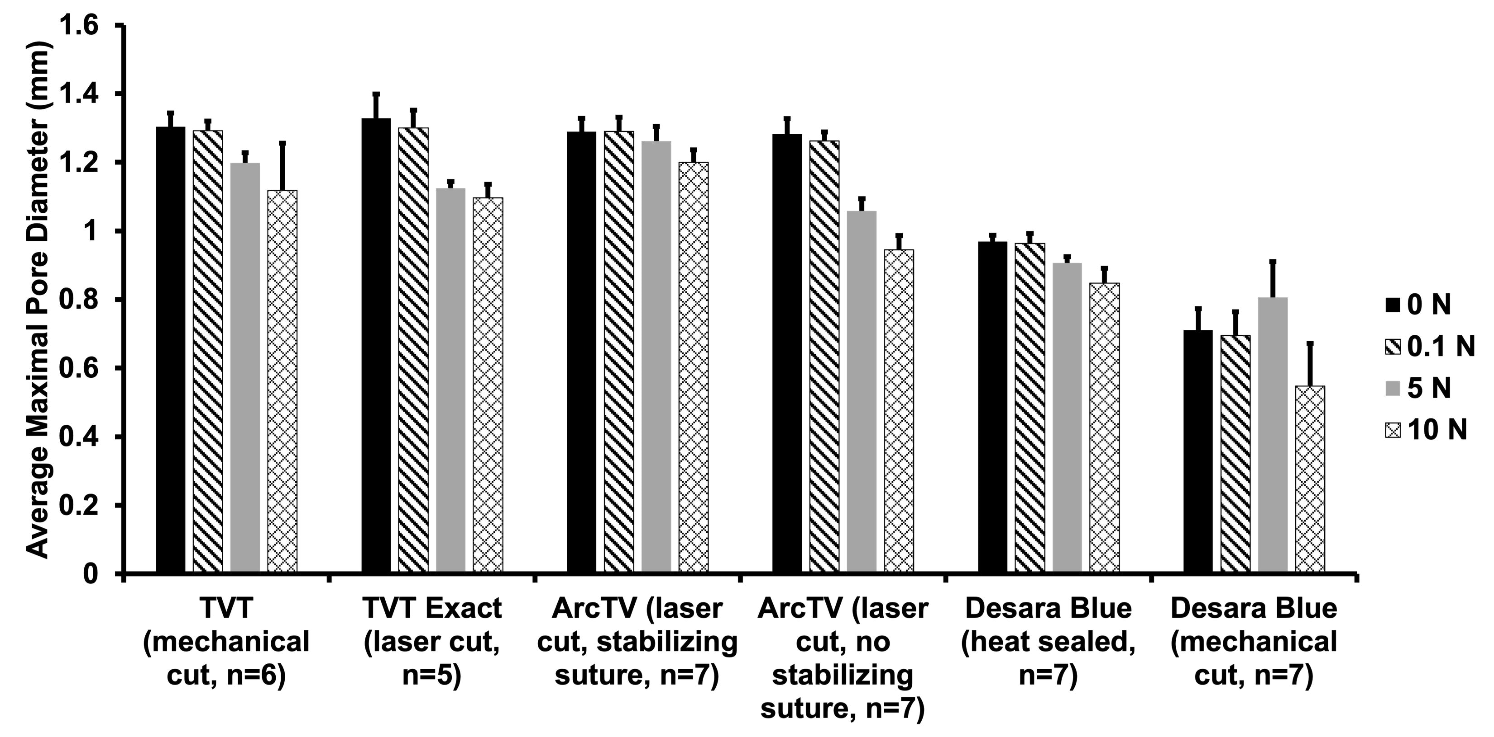
**

**Supplementary Material Figure 3.** Average maximal pore diameter following the application of 0 N, 0.1 N, 5 N, and 10 N. Overall, the maximal pore diameter decreases with increasing load. Error bars represent standard deviation.

**Supplemental Video Captions**

**Supplemental Material Video 1** -

**2 Supplemental Video Captions**

**Supplemental Material Video 1 -** Shortened video of Gynecare TVT Exact during uniaxial tensile testing. Small fibers along the edges of the MUS break away as the MUS is elongated. Note: For the purposes of this video, testing was not performed in a saline bath to prevent distortion and the video speed was increased 200%.

**Supplemental Material Video 2 -** Shortened video of TVT during uniaxial tensile testing. Small fibers along the edges of the MUS break away as the MUS is elongated (i.e., the MUS appears to unravel as it is elongated). Note: For the purposes of this video, testing was not performed in a saline bath to prevent distortion and the video speed was increased 200%.

**Supplemental Material Video 3 -** Shortened video of ArcTV without the stabilizing suturing during uniaxial tensile testing. Small fibers along the edges of the MUS break away as the MUS is elongated. Note: For the purposes of this video, testing was not performed in a saline bath to prevent distortion and the video speed was increased 200%.

**Supplemental Material Video 4 -** Shortened video of Desara Blue heat sealed during uniaxial tensile testing. Unlike the other MUSs, very few fibers were observed flying away during loading of Desara Blue heat sealed. Note: For the purposes of this video, testing was not performed in a saline bath to prevent distortion and the video speed was increased 200%.

**Supplemental Material Video 5 -** Shortened video of Desara Blue mechanical cut during uniaxial tensile testing. Small fibers along the edges of the MUS break away as the MUS is elongated. Note: For the purposes of this video, testing was not performed in a saline bath to prevent distortion and the video speed was increased 200%.
